# On the Effectiveness of Nature-Inspired Metaheuristic Algorithms for Performing Phase Equilibrium Thermodynamic Calculations

**DOI:** 10.1155/2014/374510

**Published:** 2014-05-20

**Authors:** Seif-Eddeen K. Fateen, Adrian Bonilla-Petriciolet

**Affiliations:** ^1^Department of Chemical Engineering, Cairo University, Giza 12316, Egypt; ^2^Department of Chemical Engineering, Aguascalientes Institute of Technology, 20256 Aguascalientes, AGS, Mexico

## Abstract

The search for reliable and efficient global optimization algorithms for solving phase stability and phase equilibrium problems in applied thermodynamics is an ongoing area of research. In this study, we evaluated and compared the reliability and efficiency of eight selected nature-inspired metaheuristic algorithms for solving difficult phase stability and phase equilibrium problems. These algorithms are the cuckoo search (CS), intelligent firefly (IFA), bat (BA), artificial bee colony (ABC), MAKHA, a hybrid between monkey algorithm and krill herd algorithm, covariance matrix adaptation evolution strategy (CMAES), magnetic charged system search (MCSS), and bare bones particle swarm optimization (BBPSO). The results clearly showed that CS is the most reliable of all methods as it successfully solved all thermodynamic problems tested in this study. CS proved to be a promising nature-inspired optimization method to perform applied thermodynamic calculations for process design.

## 1. Introduction

Applied thermodynamic calculations in chemical engineering often involve the repeated solution of phase stability and phase equilibrium problems as their solutions are needed during the design of several equipment and separation processes. These problems can be formulated as minimization problems, for which the global minimum represents the required result. These calculations are challenging due to the high nonlinearity of thermodynamic models used to describe the equilibrium phases, the potential nonconvexity of the thermodynamic functions used as objective, and the presence of trivial solutions in the feasible search space. Thus, the solution of this type of problems via global optimization algorithms remains to be an active area of research. These problems generally feature local minima that are comparable to the global minimum, which accentuates the need for reliable global optimizers [1, 2]. For example, the features of reactive phase equilibrium calculations increase the dimensionality and complexity of the optimization problem because the objective functions are required to satisfy the chemical equilibrium constraints [1, 2].

The global stochastic optimization methods show high probabilities to locate the global minimum within reasonable computational costs, and thus they offer a desirable balance between reliability and efficiency for finding the global optimum solution. Moreover, stochastic methods do not require any assumptions for the optimization problem at hand, are more capable of addressing the nonlinearity and nonconvexity of the objective function, and are relatively easier to program and implement, among other advantages [3].

The application of stochastic global optimization methods for solving phase equilibrium thermodynamic problems has grown considerably during last years. To date, the most popular stochastic global optimization methods have been used and applied for solving phase equilibrium thermodynamic problems, for example, simulated annealing, genetic algorithms, tabu search, differential evolution, particle swarm optimization, and ant colony optimization (ACO) [4–15]. For example, a variant of ACO was tested in the global optimization of thermodynamic problems and was found to be robust in solving vapor-liquid equilibrium parameter estimation problems [4]. Zhu et al. [5] used an enhanced simulated annealing algorithm to solve multicomponent phase stability problems. Bonilla-Petriciolet and his coworkers compared different variants of PSO [6] and different variants of simulated annealing [14] for solving phase equilibrium problems. Repulsive particle swarm optimization was also studied by Rahman et al. [8]. Rangaiah and his co-workers studied the differential evolution [9, 10], tabu search [11], and genetic algorithms [12] for solving phase stability and phase equilibrium problems.

The above studies have analyzed the capabilities and limitations of stochastic optimizers. But there exists no conclusive evaluation of those methods in comparison to one another for the solution of phase stability and phase equilibrium problems. Typically, each algorithm is introduced and compared with some of the other algorithms in a research publication. However, to the best of our knowledge, there exists no study that presents to the scientific community a ranking of the efficiency and reliability of those algorithms for the purpose of solving phase equilibrium and stability problems.

The aim of this study is provide a definitive ranking of the performance of a set of nature-inspired metaheuristic algorithms. To do so, we have selected eight of the most promising nature-inspired optimization methods based on the performance reported in the literature or obtained from our previous studies. These algorithms are cuckoo search (CS), intelligent firefly (IFA), bat (BA), artificial bee colony (ABC), monkey and krill herd hybrid (MAKHA), covariance matrix adaptation evolution strategy (CMAES), magnetic charged system search (MCSS), and bare bones particle swarm optimization (BBPSO). We systematically used those methods on some of the difficult phase stability and phase equilibrium problems reported in the literature and then analyzed their performance in terms of clear reliability and efficiency metrics.

The remainder of this paper is organized as follows. The eight optimization methods and the rationale for their selection are briefly presented in Section 2. A brief description of the phase stability and equilibrium problems is given in Section 3, including the implementation details of the eight algorithms.  Section 4 presents the results and discussion of their performance in solving these thermodynamic calculations. Finally, the conclusions of this study are summarized in Section 5.

## 2. Selection and Description of the Nature-Inspired Metaheuristic Algorithms

Each of the eight selected metaheuristics is presented below. Only brief introductions are made here. Interested readers are referred to the primary sources of those algorithms for more information.

Cuckoo search (CS) is an optimization algorithm inspired by the obligate brood parasitism of some cuckoo species by laying their eggs in the nests of other host birds [16]. Intelligent firefly algorithm (IFA) [17] is a variant of firefly algorithm [18], a metaheuristic algorithm, inspired by the flashing behavior of fireflies to attract other fireflies. MAKHA is a hybrid between monkey algorithm (MA) [19], which is inspired by the simulation of the climbing processes of monkeys to find the highest mountaintop, and krill-herd algorithm (KHA) [20], which is based on the simulation of the herding behavior of krill individuals. Covariance matrix adaptation evolution strategy (CMAES) [21] is a stochastic and derivative free method for numerical optimization of nonlinear nonconvex problems. Artificial bee colony (ABC) [22] is an optimization algorithm based on the intelligent foraging behavior of honey bee swarm. Bat algorithm (BA) [23] is another bioinspired optimization algorithm based on the echolocation behavior of microbats with varying pulse rates of emission and loudness. Magnetic charged system search (MCSS) [24] is a variant of charged system search [25], which is based on the application of physics principles such as Coulomb law and Newtonian laws of mechanics to model how charged particles affect one another during their move towards the largest bodies. In MCSS, magnetic forces are also considered in addition to electrical forces. Finally, a variant of bare bones particle swarm optimization (BBPSO) [26] is based on the original particle swarm optimization [27], but without parameters and with the incorporation of mutation and crossover operators of DE to enhance the global search capability.

Since it was not possible to include all global stochastic optimization methods available in the literature for this comparative study, a screening process was performed to select the most promising ones. This process depended mainly on the results of solving phase stability and phase equilibrium problems using global optimization methods as reported in the literature. In several publications, limited comparisons were reported between some GSO methods. For example, CMAES was selected as it was shown to perform better than shuffled complex evolution in solving phase equilibrium and phase stability problems [28]; IFA performed better than FA in general [17], CS better than integrated differential evolution [29], MCSS better than CSS for phase equilibrium and phase stability problems [30], and BBPSO better than PSO [26]. In addition, our preliminary calculations showed that MAKHA performed better than MA and KHA, and ABC and BA performed better than FA.

One approach to solving phase stability and phase equilibrium problems is to start the optimization process with a stochastic global optimizer, as the methods studied in this work. Once a certain stopping criterion is satisfied, we follow with a local optimizer, such as sequential quadratic programming, to close down to the minimum within the vicinity of the best value found by the global optimizer. This approach has been proven successful in previous studies [28–30] and it would complement any of the methods studied above. However, we restricted this study to the performance of the stochastic global optimizers without the use of a local optimizer to focus on the strength and weakness of the studied methods free from any artificial enhancement of their results.

## 3. Description of Phase Stability and Phase Equilibrium Problems Used for the Evaluation

### 3.1. Objective Functions

In this study, the phase stability and equilibrium problems are stated as a global optimization problem. Therefore, the global optimization problem to be solved is as follows: minimize *F*(**X**) with respect to *D* decision variables: **X** = (*X* ^1^, …, *X* ^*D*^). The upper and lower bounds of these variables are (*X*_max⁡_^1^,…, *X*_max⁡_^*D*^) and (*X*_min⁡_^1^,…, *X*_min⁡_^*D*^), respectively.

The phase stability, phase equilibrium, and reactive phase equilibrium calculations for testing the performance of global optimization methods are explained briefly in Table 1, which shows the problem formulation, objective function, decision variables, and constraints used for those thermodynamic calculations. Specifically, the phase stability analysis was performed using the global minimization of the tangent plane distance function (TPDF) [31], while the global optimization of the Gibbs free energy was used for phase equilibrium calculations with or without chemical reactions [2]. The mathematical formulation for phase stability and phase equilibrium calculations for nonreactive systems is an unconstrained minimization of the objective function, while the constrained Gibbs free energy minimization in reactive systems was performed using the penalty function method according to the approach reported by Bonilla-Petriciolet et al. [1]. For interested readers, several references provide a detailed description of these thermodynamic calculations [1, 2, 4, 10, 12].

Previous work reported the evaluation of global optimization methods for solving twenty-four problems [4, 28, 30]. In this work, we focused on the nine most difficult ones. The basis for the selection was the relatively lower success rates that optimization methods obtained when solving them in the previous studies. These problems are presented in Table 2.

### 3.2. Details of Numerical Implementation and Performance Metrics Used for Testing the Algorithms

All thermodynamic problems and the different optimization algorithms were coded in the MATLAB technical computing environment. The codes for CS and BA were obtained from MATLAB file exchange server as uploaded by their developers and used without change. The code for IFA was developed by the authors through minor modifications of the FA code that was obtained from the MATLAB file exchange server as well. The codes for CMAES and ABC were obtained from the developers' web sites and used without change. The code for MCSS was written by the authors based on the developer's published work [24, 25]. MAKHA was developed and coded by the authors. The code for BBPSO was obtained from its developer [26]. Each problem was solved 30 times independently and with different random initial seeds to determine the reliability of the optimization algorithms. Calculations were performed for a certain number of iterations and then stopped. This maximum value for the number of iterations was different for different algorithms. The maximum values were selected to give the same number of function evaluations at the end of the run.  Table 3 shows the values selected for the parameters of the eight optimization algorithms, which were determined using preliminary calculations.

The methods were evaluated according to the reliability and efficiency for finding the global optimum. The efficiency is determined by recording the number of function evaluations NFE for each optimization algorithm, where a low value of NFE means a higher efficiency. Note that NFE is an unbiased indicator of the computational costs required by a certain algorithm and is independent of the host hardware. In previous studies [1, 4, 6, 26, 28, 30], reliability was measured by the success rate at certain number of iterations. The success rate is defined as the ratio of number of runs in which the global minimum was attained within a tolerance at this iteration number to the total number of runs. In this work, we present a different reliability metric: a plot of the average best value against the number of function evaluations. The best values are averaged over all the runs and plotted against NFE, which is calculated at each iteration. Since the NFE needed for each iteration differs amongst the optimization methods, the plot of average best value against NFE is a better indication of reliability versus efficiency of the optimization method.

For a comparative evaluation of the global optimization methods, we have employed performance profile (PP) reported by Dolan and Moré [32], who introduced PP as a tool for evaluating and comparing the performance of optimization software. In particular, PP has been proposed to represent compactly and comprehensively the data collected from a set of solvers for a specified performance metric such as the computing time or the number of function evaluations. The PP plot allows visualization of the expected performance differences among several solvers and comparing the quality of their solutions by eliminating the bias of failures obtained in a small number of problems.

Consider *n*_*s*_ solvers (i.e., optimization methods) to be tested over a set of *n*_*p*_ problems. For each problem *p* and solver *s*, the performance metric *t*_*ps*_ must be defined. In our study, reliability of the stochastic method in accurately finding the global minimum of the objective function is considered as the principal goal, and hence the reliability performance metric is defined as

(1) $$\begin{array}{c} t_{ps}=f_{\text{calc}}-f^{*}, \end{array}$$

where f* is the known global optimum of the objective function and *f*_calc_ is the mean value of that objective function calculated by the metaheuristic over several runs. We have also used another performance metric for the evaluation of the efficiency of the method in obtaining the global minimum. This metric is the minimum number of NFE needed to reach with 10^−5^ of the global minimum.

For the performance metric of interest, the performance ratio, *r*_*ps*_, is used to compare the performance on problem *p* by solver *s* with the best performance by any solver on this problem. This performance ratio is given by

(2) $$\begin{array}{c} r_{ps}=\frac{t_{ps}}{\min\left\{t_{ps}:1\leq s\leq n_{s}\right\}}. \end{array}$$

The value of *r*_*ps*_ is 1 for the solver that performs the best on a specific problem *p*. To obtain an overall assessment of the performance of solvers on *n*_*p*_ problems, the following cumulative function for *r*_*ps*_ is used:

(3) $$\begin{array}{c} \rho_{s}\left(\zeta \right)=\frac{1}{n_{p}}\text{size}\left\{p:r_{ps}\leq \zeta \right\}, \end{array}$$

where *ρ*_*s*_(*ζ*) is the fraction of the total number of problems, for which solver *s* has a performance ratio *r*_*ps*_ within a factor of *ζ* of the best possible ratio. The PP of a solver is a plot of *ρ*_*s*_(*ζ*) versus *ζ*; it is a nondecreasing, piecewise constant function, continuous from the right at each of the breakpoints [32]. To identify the best solver, it is only necessary to compare the values of *ρ*_*s*_(*ζ*) for all solvers and to select the highest one, which is the probability that a specific solver will “win” over the rest of solvers used.

In our case, one PP plot compares how accurately the stochastic methods can find the global optimum value relative to one another, and so the term “win” refers to the stochastic method that provides the most accurate value of the global minimum in the benchmark problems used. The other PP plot compares how fast the stochastic methods can find the global minimum with a tolerance level of 10^−5^, so the term “win”, in this case, refers to the method that reaches the solution fastest for the problems used.

## 4. Results and Discussion

**Figure 1:**
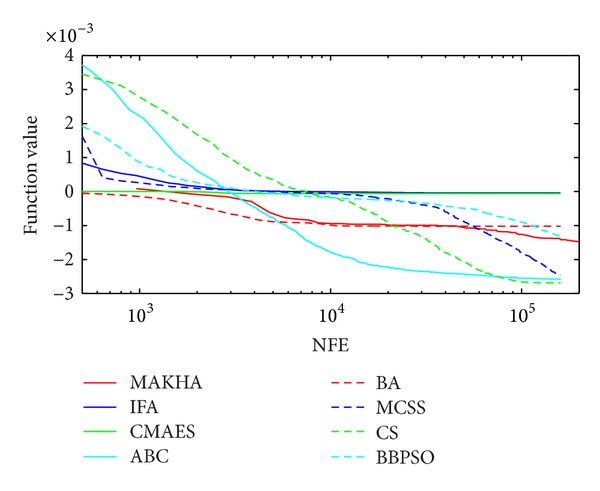
The evolution of the mean best value calculated via the eight metaheuristics versus NFE for problem T7.

**Figure 2:**
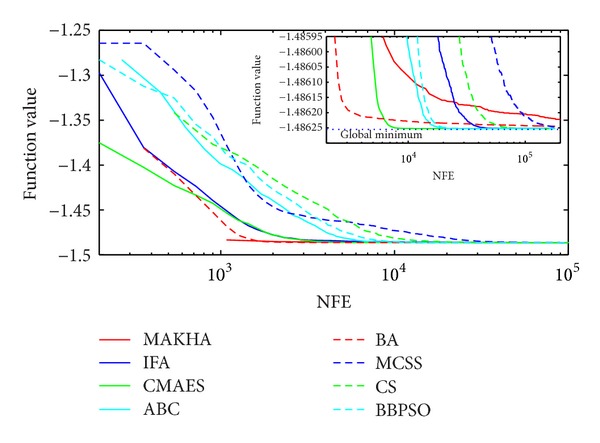
The evolution of the mean best value calculated via the eight metaheuristics versus NFE for problem T8.

**Figure 3:**
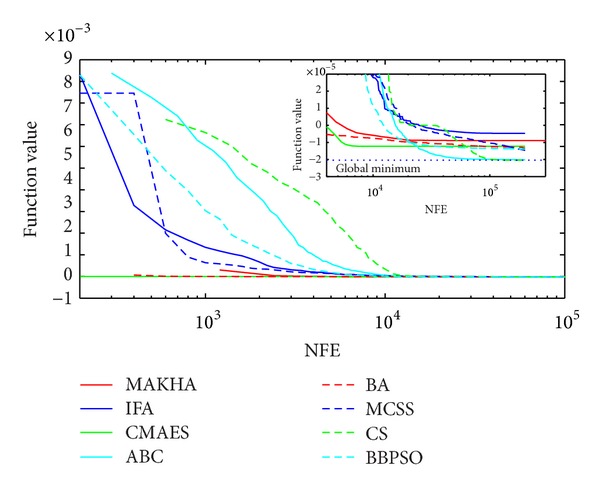
The evolution of the mean best value calculated via the eight metaheuristics versus NFE for problem T9.

**Figure 4:**
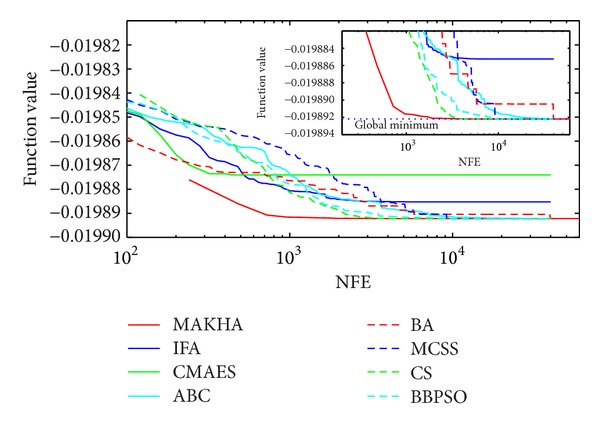
The evolution of the mean best value calculated via the eight metaheuristics versus NFE for problem G4.

**Figure 5:**
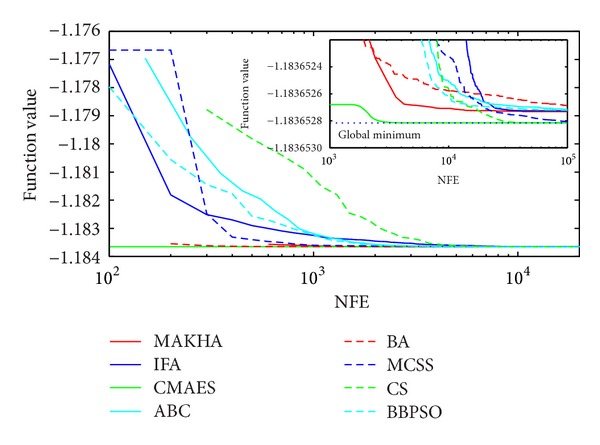
The evolution of the mean best value calculated via the eight metaheuristics versus NFE for problem G6.

**Figure 6:**
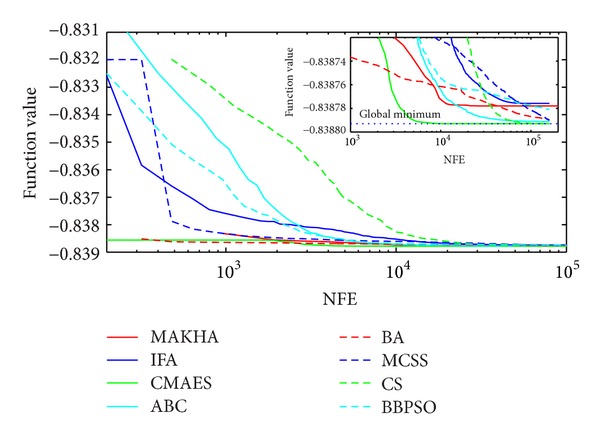
The evolution of the mean best value calculated via the eight metaheuristics versus NFE for problem G7.

**Figure 7:**
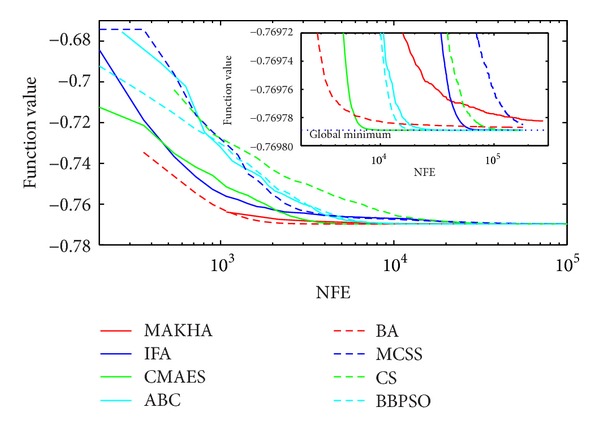
The evolution of the mean best value calculated via the eight metaheuristics versus NFE for problem G8.

**Figure 8:**
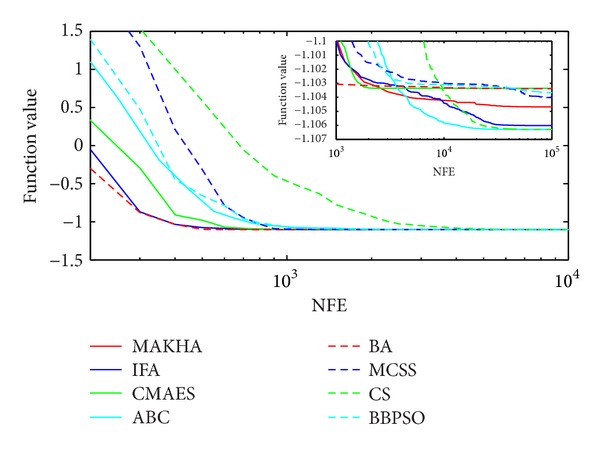
The evolution of the mean best value calculated via the eight metaheuristics versus NFE for problem RG4.

**Figure 9:**
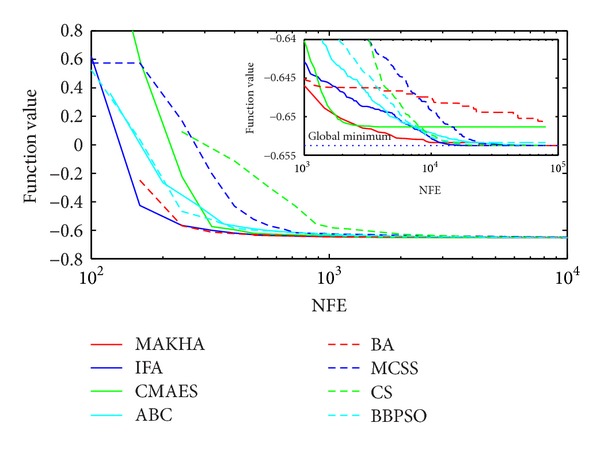
The evolution of the mean best value calculated via the eight metaheuristics versus NFE for problem RG7.

**Figure 10:**
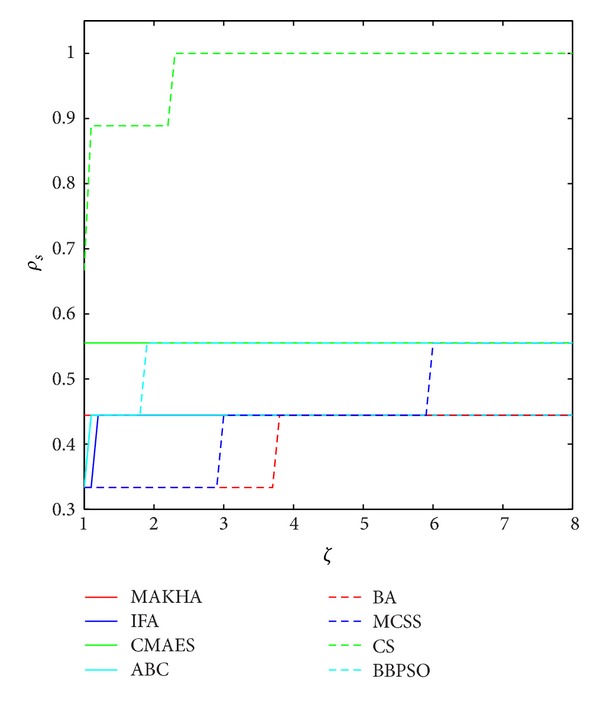
Performance profile (PP) of the reliability metric of the eightmetaheuristics for the 9 phase stability and equilibrium problems.

**Figure 11:**
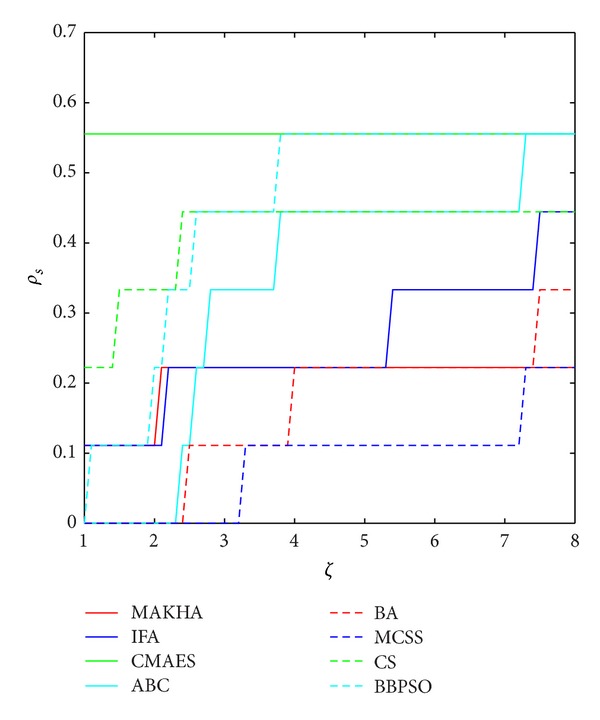
Performance profile (PP) of the efficiency metric of the eight metaheuristics for the 9 phase stability and equilibrium problems.

The results are presented in three different ways. For each problem, the mean best values are plotted versus NFE for each of the eight algorithms. These plots are found in Figures 1–9. The minimum NFE required to reach a certain tolerance from the known global minimum for each problem was calculated and presented in Table 4. The performance profiles for the reliability and efficiency metrics are shown in Figures 10 and 11, respectively. A detailed discussion of the results follows.

### 4.1. Phase Stability Problems

Problem T7 is a nine-variable phase-stability problem that is extremely difficult to solve. The means of the minimum values obtained by all methods were not close enough to the global minimum except for CS. As shown in Figure 1 and Table 4, ABC and MCSS were able to get to within 10^−3^ of the global minimum. On the other hand, CS was able to find the global minimum down to a tolerance of 10^−7^. To reach the global minimum within a tolerance of 10^−5^, it required 109280 function evaluations.

Problem T8 is also a difficult phase stability problem.  Figure 2 shows how all problems were able to reach values close to the global optimum. However, close analysis at the vicinity of the global minimum, as depicted in the inset of Figure 2, at the level of 10^−5^, revealed that MAKHA and BA failed to find the global minimum up to the end of the runs. CMAES was the most efficient as it converged to the global minimum in the least NFE by at least one order of magnitude. None of the methods was able to reach within 10^−6^ of the global minimum, as shown in Table 4.

Problem T9 is the last of the three phase stability problems. Even though, MAKHA was quite fast in approaching the global minimum, as depicted in Figure 3, it failed at converging to within 10^−5^ of the global minimum. IFA was also not able to find the global minimum. CMAES was the most efficient method in getting down to 10^−5^ distance from the global minimum but was not able to get any closer. CS, again, was the only method to converge reliably down to 10^−7^ of the global minimum.

For the phase stability problems, CS is clearly the most reliable method. It may not be as efficient in its initial approach to the global minimum as other methods such as BA or CMAES, but it outperforms the rest in terms of finding the global minimum. An open area of development for CS would be to make it more efficient via hybridization with some of the other methods in their initial approach to the global minimum.

### 4.2. Phase Equilibrium Problems

Problem G4 is a two-variable phase equilibrium problem that is relatively easy to solve. However, CMAES seemed to have been trapped in a local minimum and was unable to find its global minimum, within a tolerance of 10^−5^, as shown in Figure 4. IFA did slightly better than CMAES, but was unable to reach the global minimum within a tolerance of 10^−6^. MAKHA was the most efficient in finding the global minimum within 10^−6^ and 10^−7^, with BBPSO and CS performing quite well.

Despite the fact that CMAES was not able to solve problem G4, it was superior in solving problem G6. With only 101 NFE, CMAES reached down to 10^−6^ of the global minimum, as is shown in Figure 5. All methods converged to 10^−6^ from the global minimum, but only CMAES, CS, and MCSS converged to 10^−7^, with CMAES being ten times more efficient. This convergence pattern was repeated in problem G7. Only CMAES and CS solved the problem down to the 10^−6^ and 10^−7^ levels, with CMAES being one order of magnitude more efficient, as is clear in Figure 6 and Table 4. MAKHA, BA, and BBPSO were not able to converge at the 10^−5^ level.

Problem G8 was successfully solved at the 10^−5^ level by IFA, CMAES, ABC, BA, CS, and BBPSO, as shown in Figure 7. Only CMAES and CS solved the problem down to the 10^−7^ levels, with CMAES being one order of magnitude more efficient. In fact, CMAES was quite efficient at all tolerance levels, as shown by the NFE numbers in Table 4.

The convergence profiles of the four phase equilibrium problems (G4, G6, G7, and G8) indicated that CS is the most reliable of all algorithms as it was the only one to be able to solve all problems down to the 10^−7^ tolerance level. CMAES was the most efficient as it required one order of magnitude less NFE to solve three of the four problems down to the same tolerance level. However, CMAES failed to solve the two-variable problem that was successfully solved by all other methods, except IFA, down to the 10^−7^ level.

### 4.3. Reactive Phase Equilibrium Problems

Regardless of the number of variables, the reactive phase equilibrium problems are more difficult than the nonreactive phase equilibrium problems because the chemical reaction equilibria constraints must be satisfied. Problem R4, see Figure 8, was successfully solved down to the 10^−5^ tolerance level by CS, which was also able to converge to the global minimum at the 10^−6^ and 10^−7^ levels. MAKHA, CMAES, BA, MCSS, and BBPSO were not able to arrive even at a level of 10^−3^ from the global minimum. Similarly, CMAES and BA were not able to reach the 10^−3^ level for Problem R7. However, MAKHA, IFA, CS, and BBPSO converged down to 10^−7^ distance from the global minimum, with IFA being the most efficient down to the 10^−5^ level and BBPSO at the 10^−6^ and 10^−7^ levels.

The complete failure of CMAES to solve reactive phase equilibrium problems is remarkable. CMAES functions extremely well in certain types of problems and extremely bad in others. On the other hand, CS solved the reactive phase equilibrium problems just as it reliably solved all other problems in this study. Since CS uses Lévy walk, instead of random walk, in its global search, it can explore the search space more efficiently and avoid entrapment in local minima, as was demonstrated by our results. However, CS requires significantly large NFE to allow it to converge to the global minimum. Any attempt to improve CS performance should target its slow convergence behavior.

Our results are summarized in the PP plots of Figures 9 and 10. The reliability ranking, as extracted from the reliability PP plot of Figure 9, is as follows. CS is the most reliable, followed by CMAES, BBPSO, and MCSS, on the second level. The third level contains MAKHA, ABC, IFA, and BA, in that order. The efficiency ranking starts with CMAES, BBPSO, and ABC. The second level contains CS and IFA. The third level contains BA, MAKHA, and MCSS.

## 5. Conclusions

In this study, we have selected eight promising nature-inspired metaheuristic algorithms for the solution of nine difficult phase stability and phase equilibrium problems. These thermodynamic problems were systematically solved by the different metaheuristics and the results were tracked and compared. The results clearly show that CS is the most reliable of all tested optimization methods as it successfully solved all problems down to the 10^−5^ tolerance from the global minima. Any attempt to improve the performance of CS should target its slow convergence behavior. Recently developed CS variants [33] could provide more efficient performance for the solution of phase stability and phase equilibrium problems. These variants could be evaluated in a future study in an attempt to find the most reliable and efficient algorithm for this application. On the other hand, CMAES was the most efficient in finding the solution for the problems it was able to solve. However, it was not able to converge to the global minimum for some of the tested thermodynamic problems.

## Figures and Tables

**Table 1:** Description of thermodynamic functions and optimization problems for phase stability analysis and equilibrium calculations in reactive and nonreactive systems.

Calculation	Description	Thermodynamic function	Optimization problem
Phase stability analysis	It involves the determination of whether a system will remain in one phase at the given conditions or split into two or more phases	Tangent plane distance function $\text{TPDF}=\overset{c}{\underset{i=1}{\sum }}y_{i}\left({\mu_{i}|}_{y}-{\mu_{i}|}_{z}\right)$ where *c* is the number of components of the mixture and μ_*i*_|_*y*_ and μ_*i*_|_*z*_ are the chemical potentials calculated at trial composition *y* and feed composition *z*	$\begin{array}{c} \underset{\beta }{\min}\text{TPDF }\\ 0\leq \beta_{i}\leq 1\;i=1,\ldots ,c. \end{array}$ The decision variables are *β*_*i*_ ∈ (0,1) using the following relationships: *n*_*iy*_ = β_*i*_ *z*_*i*_ *n*_*F*_ *i* = 1,…, *c* $y_{i}=\frac{n_{iy}}{\sum_{j=1}^{c}n_{iy}}\;i=1,\ldots ,c$ where *n*_*iy*_ are the mole numbers of component *i* in phase *y* and *n*_*F*_ is the total moles in the mixture under analysis

Phase equilibrium calculation	It involves the determination of the number, type, and composition of the phases at equilibrium at the given operating conditions	Gibbs free energy of mixing (*g*) $\begin{array}{c} g=\sum_{j=1}^{\pi }\sum_{i=1}^{c}n_{ij}\ln\left(x_{ij}\gamma_{ij}\right)\\ \;\;=\sum_{j=1}^{\pi }\sum_{i=1}^{c}n_{ij}\ln\left(\frac{x_{ij}{\hat{\varphi }}_{ij}}{\varphi_{i}}\right) \end{array}$ where *π* is the number of phases at equilibrium and *θ*_*ij*_ denotes the composition (i.e., *x* or *n*) or thermodynamic property of component *i* in phase *j*	$\begin{array}{c} \underset{\beta }{\min}g\;\\ 0\leq \beta_{ij}\leq 1\;\\ i=1,\ldots ,c\;\\ j=1,\ldots ,\pi -1.\; \end{array}$ The decision variables are *β*_*ij*_ ∈ (0,1) using the following relationships: *n*_*i*1_ = β_*i*1_ *z*_*i*_ *n*_*F*_ *i* = 1, ..., *c* $\begin{array}{c} n_{ij}=\beta_{ij}\left(z_{i}n_{F}-\sum_{m=1}^{j-1}n_{im}\right)\;\\ i=1,\ldots ,c;\;j=2,\ldots ,\pi -1 \end{array}$ $n_{i\pi }=z_{i}n_{F}-\sum_{m=1}^{\pi -1}n_{im}\;i=1,...,c$

Reactive phase equilibrium Calculations	It involves the determination of the number, type and composition of the phases at equilibrium at the given operating conditions and subject to element/mass balances and chemical equilibrium constraints.	Gibbs free energy of mixing defined using reaction equilibrium constants [2] $G_{K}=g-\sum_{j=1}^{\pi }\ln K_{\text{eq}}N^{-1}n_{\text{ref},j}$ where *g* is the Gibbs free energy of mixing, ln⁡**K**_eq_ is a row vector of logarithms of chemical equilibrium constants for *r* independent reactions, **N** is an invertible, square matrix formed from the stoichiometric coefficients of a set of reference components chosen from the *r* reactions, and **n**_ref_ is a column vector of moles of each of the reference components	$\underset{n_{\mathrm{ij}}}{\min}G_{K}$ subject to $\sum_{j=1}^{\pi }\left(n_{ij}-v_{i}N^{-1}n_{\text{ref},j}\right)=n_{iF}-v_{i}N^{-1}n_{\text{ref},F}$ *i* = 1,…, *c* − *r* *n*_*ij*_ > 0 *i* = 1,…, c *j* = 1,…, π where *n*_*i*,*F*_ is the initial moles of component *i* in the feed, **v**_*i*_ is the row vector (of dimension *r*) of stoichiometric coefficients of component *i* in *r* reactions, and *n*_*ij*_ is the number of moles of component *i* in phase *j*. The constrained global optimization problem can be solved by minimizing *G*_*K*_ with respect to *c*(*π* − 1) + *r* decision variables *n*_*ij*_. In this formulation, the mass balance equations are rearranged to reduce the number of decision variables of the optimization problem and to eliminate equality constraints

**Table 2:** Details of the phase stability, phase equilibrium, and reactive phase equilibrium problems used in this study.

Code	System	Feed conditions	Thermodynamic models	Global optimum
T7	C_1_ + C_2_ + C_3_ + C_4_ + C_5_ + C_6_ + C_7–16_ + C_17+_	*n*_*F*_ = (0.7212, 0.09205, 0.04455, 0.03123, 0.01273, 0.01361, 0.07215, 0.01248) at 353 K and 38500 kPa	Phase stability problem with SRK EoS with classical mixing rules	−0.002688

T8	C_1_ + C_2_ + C_3_ + *i*C_4_ + C_4_ + *i*C_5_ + C_5_ + C_6_ + *i*C_15_	*n*_*F*_ = (0.614, 0.10259, 0.04985, 0.008989, 0.02116, 0.00722, 0.01187, 0.01435, 0.16998) at 314 K and 2010.288 kPa	Phase stability problem with SRK EoS with classical mixing rules	−1.486205

T9	C_1_ + C_2_ + C_3_ + C_4_ + C_5_ + C_6_ + C_7_ + C_8_ + C_9_ + C_10_	*n*_*F*_ = (0.6436, 0.0752, 0.0474, 0.0412, 0.0297, 0.0138, 0.0303, 0.0371, 0.0415, 0.0402) at 435.35 K and 19150 kPa	Phase stability problem with SRK EoS with classical mixing rules	−0.0000205

G4	C_1_ + H_2_S	*n*_*F*_ = (0.9813, 0.0187) at 190 K and 4053 kPa	Phase equilibrium problem with SRK EoS with classical mixing rules	−0.019892

G6	C_2_ + C_3_ + C_4_ + C_5_ + C_6_	*n*_*F*_ = (0.401, 0.293, 0.199, 0.0707, 0.0363) at 390 K and 5583 kPa	Phase equilibrium problem with SRK EoS with classical mixing rules	−1.183653

G7	C_1_ + C_2_ + C_3_ + C_4_ + C_5_ + C_6_ + C_7–16_ + C_17+_	*n*_*F*_ = (0.7212, 0.09205, 0.04455, 0.03123, 0.01273, 0.01361, 0.07215, 0.01248) at 353 K and 38500 kPa	Phase equilibrium problem with SRK EoS with classical mixing rules	−0.838783

G8	C_1_ + C_2_ + C_3_ + *i*C_4_ + C_4_ + *i*C_5_ + C_5_ + C_6_ + *i*C_15_	*n*_*F*_ = (0.614, 0.10259, 0.04985, 0.008989, 0.02116, 0.00722, 0.01187, 0.01435, 0.16998) at 314 K and 2010.288 kPa	Phase equilibrium problem with SRK EoS with classical mixing rules	−0.769772

R4	A1 + A2 *↔* A3 + A4 (1) Acetic acid (2) n-Butanol (3) Water (4) n-Butyl acetate	*n*_*F*_ = (0.3, 0.4, 0.3, 0.0) at 298.15 K and 101.325 kPa	Reactive phase equilibrium problem with UNIQUAC model and ideal gas: ln⁡*K*_eq_ = $\frac{450}{T}$ + 0.8	−1.10630

R7	A1 + A2 *↔* A3	*n*_*F*_ = (0.52, 0.48, 0.0) at 323.15 K and 101.325 kPa	Reactive phase equilibrium problem with Margules solution model: *K*_eq_ = 3.5	−0.653756

**Table 3:** Selected values of the parameters used in the implementation of the eight nature-inspired metaheuristic algorithms.

Metaheuristic	Parameter	Selected value
MAKHA	*n*	40*D*
	*B*	0.5
	*C*	−0.1
	*D*	0.1
	*D*_max⁡_	0
	*C*_*t*_	0.5
	*V*_*f*_	0.2
	*W*_*f*_	0.1

IFA	*α*_*o*_	0.5
	*β*_min⁡_	0.2
	*γ*	1
	*n*	20*D*
	*ϕ*	0.05

CMAES	*σ*	0.2
	*n*	20*D*

ABC	*n*	20*D*
	Food number	*n*/2
	limit	100

BA	*n*	20*D*
	*A*	0.25
	*r*	0.5

MCSS	CMCR	0.95
	PAR	0.1
	*N*	20*D*
	CMS	*n*/4, if integer
		*n*/2, if *n*/4 is not integer

CS	*n*	20*D*
	*p*	0.25

BBPSO	*n*	20*D*

**Table 4:** Minimum NFE for the average best value to reach 1E-3, 1E-4, 1E-5, 1E-6, and 1E-7 from the known global minimum.

Metaheuristic	*ε*	Phase equilibrium thermodynamic problem								
		T7	T8	T9	G4	G6	G7	G8	R4	R7
MAKHA	1E-3	*∞*	2164	601	121	301	481	4328	*∞*	10845
	1E*-*4	*∞*	45444	1803	121	602	4329	39493	*∞*	40006
	1E*-*5	*∞*	*∞*	*∞*	**484**	903	*∞*	*∞*	*∞*	40488
	1E*-*6	*∞*	*∞*	*∞*	2299	1806	*∞*	*∞*	*∞*	40729
	1E*-*7	*∞*	*∞*	*∞*	**2783**	*∞*	*∞*	*∞*	*∞*	41211

IFA	1E*-*3	*∞*	8820	1400	**40**	400	1600	19980	17300	**9440**
	1E*-*4	*∞*	24840	5200	**40**	3500	18880	36720	*∞*	**14480**
	1E*-*5	*∞*	39600	*∞*	1040	9000	*∞*	52560	*∞*	**20080**
	1E*-*6	*∞*	*∞*	*∞*	*∞*	15400	*∞*	73260	*∞*	25040
	1E*-*7	*∞*	*∞*	*∞*	*∞*	*∞*	*∞*	*∞*	*∞*	30160

CMAES	1E*-*3	*∞*	3961	**201**	41	**101**	**161**	3961	*∞*	*∞*
	1E*-*4	*∞*	5761	**201**	41	**101**	**2721**	5401	*∞*	*∞*
	1E*-*5	*∞*	**7381**	**5801**	*∞*	**101**	**4801**	**7021**	*∞*	*∞*
	1E*-*6	*∞*	*∞*	*∞*	*∞*	**101**	**8641**	**8821**	*∞*	*∞*
	1E*-*7	*∞*	*∞*	*∞*	*∞*	**2401**	**11681**	**10621**	*∞*	*∞*

ABC	1E*-*3	**9840**	8010	4100	60	850	2480	6570	**9750**	9560
	1E*-*4	*∞*	12150	9700	60	1950	7920	11790	**24959**	48236
	1E*-*5	*∞*	17010	21500	1220	3850	34644	19530	*∞*	*∞*
	1E*-*6	*∞*	*∞*	**58104**	12460	8650	*∞*	27990	*∞*	*∞*
	1E*-*7	*∞*	*∞*	*∞*	33092	*∞*	*∞*	41850	*∞*	*∞*

BA	1E*-*3	*∞*	**1980**	400	80	200	320	**1800**	*∞*	*∞*
	1E*-*4	*∞*	**2340**	400	80	200	5760	**3240**	*∞*	*∞*
	1E*-*5	*∞*	*∞*	43200	6960	400	*∞*	16920	*∞*	*∞*
	1E*-*6	*∞*	*∞*	*∞*	8400	3700	*∞*	*∞*	*∞*	*∞*
	1E*-*7	*∞*	*∞*	*∞*	8440	*∞*	*∞*	*∞*	*∞*	*∞*

MCSS	1E*-*3	87520	35640	800	**40**	400	640	26460	*∞*	21200
	1E*-*4	*∞*	80820	6200	**40**	1100	36800	104400	*∞*	34160
	1E*-*5	*∞*	178920	78000	3520	3300	157600	*∞*	*∞*	64320
	1E*-*6	*∞*	*∞*	*∞*	7880	11000	*∞*	*∞*	*∞*	*∞*
	1E*-*7	*∞*	*∞*	*∞*	9120	49600	*∞*	*∞*	*∞*	*∞*

CS	1E*-*3	41440	17460	7400	120	1700	7840	22140	16100	10640
	1E*-*4	**79200**	35100	11800	120	4100	26400	44820	30900	17680
	1E*-*5	**109280**	61380	57400	1160	5500	52960	74700	**50300**	30000
	1E*-*6	**132640**	*∞*	93400	3480	6700	93280	112500	**70300**	39760
	1E*-*7	**155040**	*∞*	**131400**	4440	24100	140000	152820	**87300**	53840

BBPSO	1E*-*3	*∞*	9900	2800	**40**	600	1760	7380	*∞*	**9440**
	1E*-*4	*∞*	14040	7400	**40**	1700	12640	11340	*∞*	17920
	1E*-*5	*∞*	18720	21600	920	3300	*∞*	15300	*∞*	21440
	1E*-*6	*∞*	*∞*	*∞*	**2200**	6000	*∞*	19080	*∞*	**23360**
	1E*-*7	*∞*	*∞*	*∞*	5120	*∞*	*∞*	22500	*∞*	**28400**

## Nomenclature

*A*: Parameter used in BA
ABC: Artificial bee colony
*B*: Parameter used in MAKHA algorithm
BA: Bat algorithm
BBPSO: Bare bones particle swarm optimization
*C*: Parameter used in MAKHA algorithm
CMAES: Covariance matrix adaptation evolution strategy
CMCR: Parameter used in MCSS
CMS: Parameter used in MCSS
CS: Cuckoo search
*C*_*t*_: Empirical and experimental constants—used in MAKHA
*D*: Dimension of the problem, Parameter used in MAKHA Algorithm
*D*_max⁡_: Maximum diffusion speed—used in MAKHA
*F*, *F*_obj_: Objective function
FA: Firefly algorithm
*G*: Gibbs free energy
**g***: Global minimum
*I*: A counter
*i*, *j*: Index of the component, or index used in an algorithm
IFA: Intelligent firefly algorithm
*K*_eq_: Equilibrium constant
*m*: Dimension of the problem, that is, number of variables
MAKHA: Monkey Algorithm-Krill Herd Algorithm hybrid
MCSS: Magnetic charged system search
*N*: The number of iterations (criterion maximum number)
*n*: Population number
NFE: Number of function evaluations
*N*_max⁡_: Maximum induced speed
*N*_*P*_: Population size (number of points)
*n*_*p*_: Number of problems
**n**_ref_: Column vector of moles of each of the reference components
*n*_*s*_: Number of solvers
NV: Dimension of the problem, that is, number of variables
*p*: Parameter used in CS
PP: Performance profile
*R*: The half-range of boundaries between the lower boundary and the upper boundary of the decision variables (*X*)—used in MAKHA
*r*: Parameter used in BA
*r*_*ps*_: The performance ratio
TPDF: Tangent plane distance function
*t*_*ps*_: Performance metric
*V*_*f*_: Foraging speed—used in MAKHA
*w*_*f*_: Inertia weight—used in MAKHA
*X*: Decision variables
*x*: Decision variables
*Y*: Decision variables
*y*: Decision variables
*c*: Number of components.

## Greek Letters

*ζ*: The simulating value of *r*_*ps*_
*ς*: The counter of *ρ* points
*ζ*_max⁡_: The maximum assumed value of *r*_*ps*_
*β*: Transformed decision variables used instead of mole fractions
*ε*: Vector of random numbers in FA
*ϕ*: Fugacity coefficient, Fraction of top fireflies to be utilized in the move—Parameter used in IFA
*γ*: Activity coefficient, Parameter used in IFA
*π*: Number of phases
*μ*: Chemical potential
*α*_*o*_: Parameter used in IFA
*β*_*o*_: Parameter used in IFA
*ρ*: The cumulative probabilistic function of *r*_*ps*_ and the fraction of the total number of problems
*σ*: Parameter used in CMAES.

## Subscripts

*F*: Feed
*I*: Index for the components in the mixture
min: Minimum value
*O*: Initial value of the parameter
*y*: At composition *y*
*z*: At composition *z*.

## Superscripts

0: Pure component.

## References

[1] Bonilla-Petriciolet A, Rangaiah GP, Segovia-Hernández JG (2011). Constrained and unconstrained Gibbs free energy minimization in reactive systems using genetic algorithm and differential evolution with tabu list. *Fluid Phase Equilibria*.

[2] Zhang H, Bonilla-Petriciolet A, Rangaiah GP (2011). A review on global optimization methods for phase equilibrium modeling and calculations. *The Open Thermodynamics Journal*.

[3] Rangaiah GP (2010). *Stochastic Global Optimization: Techniques and Applications in Chemical Engineering*.

[4] Fernández-Vargas JA, Bonilla-Petriciolet A, Segovia-Hernández JG (2013). An improved Ant Colony Optimization method and its application for the thermodynamic modeling of phase equilibrium. *Fluid Phase Equilibria*.

[5] Zhu Y, Wen H, Xu Z (2000). Global stability analysis and phase equilibrium calculations at high pressures using the enhanced simulated annealing algorithm. *Chemical Engineering Science*.

[6] Bonilla-Petriciolet A, Segovia-Hernández JG (2010). A comparative study of particle swarm optimization and its variants for phase stability and equilibrium calculations in multicomponent reactive and non-reactive systems. *Fluid Phase Equilibria*.

[7] Bonilla-Petriciolet A, Rangaiah GP, Segovia-Hernández JG, Jaime-Leal JE (2010). Stochastic global optimization: techniques and applications in chemical engineering. *World Scientific*.

[8] Rahman I, Das AK, Mankar RB, Kulkarni BD (2009). Evaluation of repulsive particle swarm method for phase equilibrium and phase stability problems. *Fluid Phase Equilibria*.

[9] Srinivas M, Rangaiah GP (2007). Differential evolution with tabu list for global optimization and its application to phase equilibrium and parameter estimation problems. *Industrial and Engineering Chemistry Research*.

[10] Srinivas M, Rangaiah GP (2007). A study of differential evolution and tabu search for benchmark, phase equilibrium and phase stability problems. *Computers and Chemical Engineering*.

[11] Teh YS, Rangaiah GP (2003). Tabu search for global optimization of continuous functions with application to phase equilibrium calculations. *Computers and Chemical Engineering*.

[12] Rangaiah GP (2001). Evaluation of genetic algorithms and simulated annealing for phase equilibrium and stability problems. *Fluid Phase Equilibria*.

[13] Nagatani G, Ferrari J, Cardozo Filho L (2008). Phase stability analysis of liquid-liquid equilibrium with stochastic methods. *Brazilian Journal of Chemical Engineering*.

[14] Bonilla-Petriciolet A, Vázquez-Román R, Iglesias-Silva GA, Hall KR (2006). Performance of stochastic global optimization methods in the calculation of phase stability analyses for nonreactive and reactive mixtures. *Industrial and Engineering Chemistry Research*.

[15] Corazza ML, Cardozo Filho L, Vladmir Oliveira J, Dariva C (2004). A robust strategy for SVL equilibrium calculations at high pressures. *Fluid Phase Equilibria*.

[16] Yang X-S, Deb S Cuckoo search via Lévy flights.

[17] Fateen SEK, Bonilla-Petriciolet A (2014). Intelligent firefly algorithm for global optimization. *Studies in Computational Intelligence*.

[18] Yang XS (2007). Firefly algorithm. *Nature-Inspired Metaheuristic Algorithms*.

[19] Zhao R, Tang W (2008). Monkey algorithm for global numerical optimization. *Journal of Uncertain Systems*.

[20] Gandomi AH, Alavi AH (2012). Krill herd: a new bio-inspired optimization algorithm. *Communications in Nonlinear Science and Numerical Simulation*.

[21] Hansen N, Müller SD, Koumoutsakos P (2003). Reducing the time complexity of the derandomized evolution strategy with covariance matrix adaptation (CMA-ES). *Evolutionary Computation*.

[22] Karaboga D, Basturk B (2007). A powerful and efficient algorithm for numerical function optimization: artificial bee colony (ABC) algorithm. *Journal of Global Optimization*.

[23] Yang X-S (2010). A new metaheuristic Bat-inspired algorithm. *Studies in Computational Intelligence*.

[24] Kaveh A, Share MAM, Moslehi M (2013). Magnetic charged system search: a new meta-heuristic algorithm for optimization. *Acta Mechanica*.

[25] Kaveh A, Talatahari S (2010). A novel heuristic optimization method: charged system search. *Acta Mechanica*.

[26] Zhang H, Kennedy DD, Rangaiah GP, Bonilla-Petriciolet A (2011). Novel bare-bones particle swarm optimization and its performance for modeling vapor-liquid equilibrium data. *Fluid Phase Equilibria*.

[27] Kennedy J, Eberhart R Particle swarm optimization.

[28] Fateen S-EK, Bonilla-Petriciolet A, Rangaiah GP (2012). Evaluation of covariance matrix adaptation evolution strategy, shuffled complex evolution and firefly algorithms for phase stability, phase equilibrium and chemical equilibrium problems. *Chemical Engineering Research and Design*.

[29] Bhargava V, Fateen SEK, Bonilla-Petriciolet A (2013). Cuckoo Search: a new nature-inspired optimization method for phase equilibrium calculations. *Fluid Phase Equilibria*.

[30] Elnabawy AO, Fateen SEK, Bonilla-Petriciolet A (2014). Phase stability analysis and phase equilibrium calculations in reactive and non-reactive systems using Charged System Search algorithms. *Industrial & Engineering Chemistry Research*.

[31] Michelsen ML (1982). The isothermal flash problem. Part I. Stability. *Fluid Phase Equilibria*.

[32] Dolan ED, Moré JJ (2002). Benchmarking optimization software with performance profiles. *Mathematical Programming B*.

[33] Fister IFD, Fister I (2013). A comprehensive review of cuckoo search: variants and hybrids. *International Journal of Mathematical Modelling and Numerical Optimisation*.

